# Indoor Volatile Organic Compounds and Chemical Sensitivity Reactions

**DOI:** 10.1155/2013/623812

**Published:** 2013-10-21

**Authors:** Tin-Tin Win-Shwe, Hidekazu Fujimaki, Keiichi Arashidani, Naoki Kunugita

**Affiliations:** ^1^Center for Environmental Health Sciences, National Institute for Environmental Studies, Tsukuba, Ibaraki 305-8506, Japan; ^2^Center for Environmental Risk Research, National Institute for Environmental Studies, Tsukuba, Ibaraki 305-8506, Japan; ^3^University of Occupational and Environmental Health, 1-1 Iseigaoka, Yahatanishi-ku, Kitakyushu, Fukuoka 807-8555, Japan; ^4^Department of Environmental Health, National Institute of Public Health, 2-3-6 Minami, Wako City, Saitama 351-0197, Japan

## Abstract

Studies of unexplained symptoms observed in chemically sensitive subjects have increased the awareness of the relationship between neurological and immunological diseases due to exposure to volatile organic compounds (VOCs). However, there is no direct evidence that links exposure to low doses of VOCs and neurological and immunological dysfunction. We review animal model data to clarify the role of VOCs in neuroimmune interactions and discuss our recent studies that show a relationship between chronic exposure of C3H mice to low levels of formaldehyde and the induction of neural and immune dysfunction. We also consider the possible mechanisms by which VOC exposure can induce the symptoms presenting in patients with a multiple chemical sensitivity.

## 1. Introduction

Indoor pollutants sometimes induce health problems. The increase in neurological symptoms and immunological abnormalities in some sensitive populations that live in buildings with relatively high concentrations of volatile organic compounds (VOCs) has been recognized as the sick building syndrome (SBS) or multiple chemical sensitivity (MCS). Recent prevalent research suggests that MCS has a physiological and not a psychological etiology [1]. The features of chemical sensitivity overlap with those of addiction, allergy, and toxicity [2]. Because the induction of MCS might be related to VOCs in houses and offices, there may be a relationship between immunological and neurological abnormalities and inhalation of VOCs. Plausible mechanisms of action to explain MCS and its susceptible subpopulations are lacking. However, there is rapidly accumulating evidence that environmental agents can modify health by disrupting the homeostatic mechanisms that regulate the nervous, endocrine, and immune systems [3]. 

Neurogenic inflammation can be induced by air pollutants [4], and exposure to either an allergen or chemical irritant leads to a sensory nerve impulse. Chemical irritants directly trigger peripheral nerve receptors. Once the impulse reaches the central nervous system (CNS), it is redirected to another peripheral location leading to the release of neuropeptides that produce inflammation at the second site [5]. Neurogenic switching explains how antigen, stress, chemical exposure, or damage at one body site might lead to diverse symptoms at multiple distant sites. Sensory neuron sensitivity to irritants and its interactions with airway tissues and other peripheral tissues may be related to neurogenic disorders. Toxic components and related compounds in air pollutants can be transported from the nasal mucosa to the olfactory bulb [6]. Therefore, we speculate that VOCs as indoor pollutants can stimulate the sensory nerve terminal and olfactory system release of neuropeptides and cytokines that modulate neuroimmune interactions. Neurotoxic reactions to air pollutants may secondarily result from the distraction caused by sensory stimulation [7]. Common chemical sense irritancy refers to sensations that arise from stimulation of the trigeminal nerve endings in the skin and mucosal surfaces of the head [8]. Primary irritant gases, such as formaldehyde (FA), may evoke pungent sensations at subtoxic exposures. Subsequently, local nerves activate local reflexes, such as the neurogenic reflex, whereby impulses transmitting toward the CNS produce activity in the periphery along unstimulated branches. This leads to the release of possible mediators of irritation, such as substance P, or to impulses traveling to more remote sites that induce reflexes such as momentary dyspnea or cough [7].

MCS is an amplification of the nonspecific immune response to low-level irritants in the upper airways [9]. Incidence of immunological symptoms such as asthma or wheezing, susceptible to infections, increased in MCS patients. It is suspected that exposure to low levels of indoor air pollutants modulates allergic and neurogenic inflammation [10]. In high-responder rats, the cholinergic-response rate increases upon exposure to organophosphate; however, there are no appropriate animal models that describe the mechanisms of MCS. FA is a toxic indoor air pollutant derived from furniture and construction materials that is found at relatively high concentrations in indoor environments [11, 12]. In this review, we focus on the effects of low-level FA inhalation on neuronal and immunological parameters and discuss the relationship between MCS and FA inhalation. Although chronic exposure to FA is not known to damage neurological functions, this may be due to a lack of study.

## 2. Effect of Long-Term Low-Level FA Exposure on the Mouse Olfactory System

MCS is a disease of unknown etiology. It has been hypothesized that MCS occurs from repeated exposure to low levels of chemicals, particularly VOCs; therefore, the olfactory system probably plays an important role in the initial expression of MCS symptoms [13, 14]. Olfactory stimulation probably represents the most likely route of exposure [15], and it is reasonable to assume that a significant activation of the olfactory epithelium (OE) cells can provide sufficient input into the CNS limbic circuits to induce sensitization. There may be a mechanism by which repeated olfactory stimulation and other sensitization processes induced by some other physiological process are exacerbated by prior, repeated olfactory stimulation. The olfactory system consists of the OE, the main olfactory bulb (MOB), and higher brain centers such as the piriform cortex and the amygdala. FA is aversive to mice at concentrations that approximate sensory irritation in humans [16].

In our previous study, morphological analysis of the mouse olfactory system was conducted after a long-term exposure to low-level FA to examine the association between olfactory function and the induction of MCS [17]. In that study, the mice were divided into four groups and exposed to 0, 80, 400, or 2000 ppb of FA vapor. After a long-term exposure (3 months), the mice were anesthetized and perfused with fixative solutions. The OE and the brains were removed and processed for morphological analysis. When the ultrastructure of the OE surface was examined in mice exposed to 2000 ppb of FA vapor, there were a slight degeneration and injury of microvilli of the supporting cell in several mice; however, most of the mice had no characteristic OE degeneration. Histological and immunocytochemical analyses of the OE indicated that there was no difference in the thickness and the expression of the olfactory marker protein (a marker of mature olfactory neurons) between the mice exposed to FA and control mice. Our results indicated that the long-term exposure to low-level FA induced no severe effect on the OE and that the OE was still functionally capable of detecting and transmitting olfactory information. We also found that there was no significant difference of olfactory bulb size between the control group and FA exposed groups [17]. 

Tyrosine hydroxylase (TH), which initiates dopamine synthesis, is abundant in periglomerular (PG) cells in the MOB, and its expression is a useful marker for monitoring olfactory function [18, 19]. Immunocytochemical analysis of TH-positive PG cells showed that long-term exposure to low-level FA induces an increase in the number of TH-expressing PG cells [17]. These results suggest that MOB activity is modulated by long-term exposure to low levels of FA.

The axons of olfactory neurons form synaptic contacts with the dendrites of secondary neurons (mitral and PG cells) in the olfactory bulb glomerulus, and it is well known that a change in the size of a synapse is activity dependent. Results of an electron microscopic analysis revealed a decrease in the size of a synapse in mice subjected to a long-term exposure of low levels of FA. Further studies are necessary to clarify the biological significance of the synaptic changes in the present study. 

In the central olfactory system, MOB neurons mainly project to the piriform cortex and amygdala. In these areas, gamma-aminobutyric acidergic (GABAergic) inhibitory neurons are colocalized with the calcium-binding proteins, calbindin and parvalbumin [20]. Our preliminary results indicated that a long-term exposure to low levels of FA induced an increase in the number of parvalbumin and calbindin expressing cells in the amygdale (unpublished results). These results suggest that long-term exposure to a low level of FA modulates the GABA inhibitory system in the central olfactory areas. The functions of the amygdala are variable but include the emotional, motivational, and homeostatic functions of the brain [21]. The piriform cortex is a higher center for processing of odor sensory information, especially odor memory [22]. It may be that these brain functions are altered by repetitive, low doses of FA. By the repetition of low-dose chemical stimulation via olfactory bulb, abnormal response in signal pathway of central nervous system induced the persistence of activation. 

The results of this morphological study indicate that the olfactory system is influenced by a long-term exposure to low levels of FA. Physiological and behavioral analyses are necessary to clarify the relationship between MCS and the olfactory system.

## 3. Estimation of the Effect of FA and Toluene Inhalation on the HPA Axis

The hypothalamo-pituitary-adrenal gland (HPA) axis responds to stress by initiating a cascade of endocrine events including the hypothalamic secretion of corticotrophin releasing hormone (CRH), the release of adrenocorticotropic hormone (ACTH) from the anterior pituitary, and the secretion of corticosteroids from the adrenal gland [23–26]. Activation of the HPA axis by stress is dependent on the characteristics of the stressor. We examined the effects of FA inhalation on the HPA axis in female mice. Our study showed that long-term exposure to low levels of FA and the allergic condition induced by ovalbumin (OVA) sensitization may act as an HPA axis stressor [27]. Briefly, we discovered a dose-dependent upregulation (0, 80, 400, and 2000 ppb) of the number of CRH-immunoreactive (ir) neurons and ACTH-ir cells and ACTH mRNA expression in nonallergenic mice exposed to FA. Furthermore, the allergic reaction and exposure to FA acted in a synergic manner on the hypothalamus and pituitary gland. These values were all significantly higher (*P* < 0.05) in control (unexposed) allergenic mice than in control nonallergenic mice. These values were significantly higher in the 80 ppb allergenic group mice compared with the 80 ppb nonallergenic mice but significantly less in the 2000 ppb allergenic mice than in the nonallergenic mice, suggesting that the 2000 ppb allergenic group mice suffered from impaired HPA axis function. The appearance of SBS in humans may result from a depressed HPA axis that is unable to react to the secondary stress (headache, mental fatigue, nausea, etc.) induced by FA [28]. Therefore, there may be a synergistic effect between low-level FA-induced and antigen-stimulated lesions of the hypothalamus and pituitary gland.

We used toluene as a chemical stressor to study whether these effects of FA exposure on the HPA axis are specific to FA. Toluene is an organic solvent that is widely used in industrial glues, lacquers, and paint removers and induces MCS in humans [29–31]. The toluene inhalation experiment consisted of two related studies. To mimic the preinhalation trigger produced by high-dose toluene, mice were exposed to 500 ppm toluene for three consecutive days prior to FA inhalation [32]. After the three days, groups of mice were exposed to various doses of (0, 80, 400, and 2000 ppb) FA as before [33]. The results showed that the number of CRH-ir neurons, the proportion of ACTH-ir cells, and ACTH mRNA expression were upregulated according to the inhaled dose of FA. The same results were found for the number of ACTH cells with the exception that the number of ACTH cells in the 2000 ppb subgroup was reduced compared with the 400 ppb subgroup. We previously described that abundant dilatation of the sinusoids was found in the anterior pituitary of the 80, 400, and especially the 2000 ppb subgroups compared with the control group [33]. Although the proportion of ACTH cells increased with the dose of FA inhaled, these results explain the reduction in the number of ACTH cells in the 2000 ppb subgroup. 

To compare the effect of FA inhalation with that of toluene, each group was further divided into control and 50 ppm subgroups of 0 and 50 ppm with and without OVA sensitization [33]. ACTH mRNA expression in the 50 ppm toluene subgroup of nonallergenic mice was greater than in the control nonallergenic group, and ACTH mRNA expression in the control allergenic group was greater than in the control nonallergenic group. Furthermore, the ACTH mRNA expression in the 50 ppm toluene subgroup of allergenic mice was greater than in the control allergenic mice. We are currently investigating the number of hypothalamic CRH-ir cells and the proportion and number of pituitary ACTH-ir cells in these mice. It follows that the inhalation of low-level toluene may have the same effect on the HPA axis as FA, with the exception that there was an increase in number and size of the sinusoids in the anterior pituitary of mice that inhaled toluene. 

## 4. Effect of Low-Level FA Inhalation on the Expression of Neurotransmitter mRNAs in the Mouse Brain

Patients suffering from MCS report hypersensitivity to a wide variety of environmental chemicals including VOCs. Because most of the common symptoms are extreme fatigue, headache, gastrointestinal problems, depression, anxiety, irritability, and sensitivity to perfumes, it can be postulated that these symptoms accompany brain dysfunction. Moreover, recent studies indicate that neural plasticity is involved in the initiation and the development of MCS [34]. This kindling causes deterioration of long-term potentiation, which is a mechanism of efficient learning and memory. Glutamate-responsive neurons are common in the limbic system, a site of short-term memory and associated functions, and glutamate levels correlate with kindling [35]. When toxic chemicals are bound to the GABA*α* receptor, their response to glutamate is disinhibited.

Thus, we studied the effect of low-level FA exposure, which is highly suspected to initiate MCS and its symptoms, on the expression of neural transmission-related mRNAs in the mouse brain (400 ppb, daily for 16 h, 5 days per week for 12 weeks). Semiquantification of mRNAs by RT-PCR revealed that FA exposure caused an increase in the glutamate receptor epsilon 1 subunit mRNA in the neocortex and hippocampus and in the epsilon-1 and epsilon-2 mRNA in the amygdala [36]. Exposure to FA also decreased the epsilon-2 mRNA in the neocortex and hippocampus. In the hypothalamus, FA increased 5-hydroxytriptamine 1A-receptor mRNA and GABA receptor *α*-1 subunit mRNA. The continuous disruption of GABA and unstable ACTH action through the synergistic effects of indoor chemical pollutants lead to unanticipated threshold changes, time-dependent sensitization [37] and may cause kindling.

## 5. Immunological Alteration in Mice Exposed to Low Levels of FA

Exposure to FA elicits a variety of allergic signs and symptoms and irritates the upper respiratory tract. There is a positive relationship between worker exposure to FA and asthma or asthmatic bronchitis [38, 39]. Long-term exposure to FA significantly increases the numbers of CD26-IL-2-positive cells, B cells, and autoantibodies in some patients with multiple-organ symptoms that involve the CNS, upper- and lower-respiratory systems, and gastrointestinal tract [40]. An increase in the CD4/CD8 ratio, due to a low percentage of CD8 positive cells, has also been observed among MCS patients [41]. However, another study found no indication of immunologically mediated respiratory disease in a group of workers exposed to FA, but some appeared to experience respiratory or ocular symptoms caused by an irritant mechanism [42].

Exposure to low levels of FA affects various immune functions in animals. In guinea pigs, an 8 h exposure to 300 ppb FA increased airway reactivity to acetylcholine [43], and, after five consecutive days of exposure to 250 ppb FA, OVA sensitivity was significantly enhanced, but there was no effect of a 130 ppb exposure [44]. Exposure to 1600 ppb FA for 10 days increased OVA-specific IgE production in mice intranasally sensitized with OVA [45]. Therefore, short-term FA exposure may directly enhance sensitization in airways and aggravate allergic inflammatory reactions. However, the long-term effects of low-level FA exposure on allergic inflammation are largely unknown. We tried to identify the presence or absence of hypersensitivity reactions in mice exposed to low doses of FA. However, exposure of mice to 80 or 400 ppb FA alone resulted in no significant changes in proinflammatory cytokine production in bronchoalveolar lavage fluid and in the hippocampus, total antibody production and lymphocyte subpopulations in peripheral blood, or infiltration of inflammatory cells to the lung; however, OVA-immunized mice exposed to 2000 ppb FA had significantly more inflammatory cells in the bronchoalveolar lavage fluid.

Exposure to FA significantly increased hippocampal nerve-growth factor (NGF) mRNA (80 and 400 ppb) and NGF content (400 ppb) in OVA-immunized mice [46]. In contrast, low levels of FA may act in concert with OVA stimulation to suppress NGF production in the plasma and bronchoalveolar lavage fluid [47]. Neurotrophins, including NGF, and brain-derived neurotrophic factors play an important role in the development of airway inflammation and hyperresponsiveness by inducing the production of tachykinins, such as substance P, which are involved in allergic responses [47]. It is likely that exposure to FA stimulates vagus nerve endings to release neuropeptides that can activate immunocompetent cells and modulate allergic inflammation. Immunological inflammation is induced at the local site by invading antigenic substances or chemicals, whereas neurogenic inflammation is induced at sites distant from stimulation. Although FA is a potent contact allergen, it lacks the ability to cause sensitization of the respiratory tract [48]. Our data suggest that exposure to FA disrupts the regulatory mechanisms of neurogenic and immunological inflammation that contribute to host homeostasis. In animal models, neurogenic inflammation may contribute to the inflammatory response to allergens, whereas chronic inflammation, which causes the release of neurotrophins from inflammatory cells, may lead to changes in innervation patterns [49].

## 6. Modulation of Neuroimmune Response in Mice Exposed to FA

Recently, an investigation of the effect of repeated low-level FA on escape or cocaine-induced behavior in mice showed that 1000 ppb FA inhalation is aversive and produces behavioral changes [16, 50]. FA inhalation induces tachykinin release from sensory nerve endings in rats [51]. Sneezing can be provoked by FA and can be evoked from C-fiber stimulation [52]. We found that sneezing frequency in mice dose dependently increased by FA inhalation [53]. Because we have shown the influence of long-term exposure to low levels of FA in the olfactory neurons and in the Ca-binding proteins in the amygdala and piriform cortex, low-level FA exposure may stimulate olfactory and trigeminal pathways and result in an abnormal response of CNS signaling pathways.

Concerning the roles of substance P, an increase in the number of immunoreactive nerve fibers was found in asthmatics compared to nonasthmatics, but less immunoreactivity was found in lung tissues of the asthmatics patients [54]. Short-term inhalation of irritants exacerbates allergic inflammation due to the release of substance P and related neuropeptides. However, the contribution of persistent inhalation of low-level irritant agents to neuroimmune inflammation remains unknown. FA can activate the sensory irritant receptor by reacting with a thiol group in the receptor and produce a more potent effect than substances physically adsorbed to the receptor [52]. During toluene diisocyanate (TDI) exposure, TDI-induced release of NGF may mediate substance P upregulation in airway-sensory neurons [55]. Not only the production of NGF but also the release of substance P in CNS and immunogenic inflammation were also modulated by low-level FA as well as TDI exposure.

Because ACTH suppresses the immune system [56], chronic stress can modulate the HPA axis and alter the innate and acquired immune response to infection [57]. CRH release from the hypothalamus stimulates the production of neuropeptides that irritate sensory organs [58], and activation of the HPA axis in FA-exposed mice directly and indirectly affects innate and acquired immune responses in the brain.

A proposed central mechanism of MCS induced by organic solvents involves the widespread stimulation of NMDA activity in the limbic system followed by a widespread increase in nitric oxide and peroxynitrite [59]. Our results stating that low levels of FA increase hippocampal NMDA-mRNA expression are in agreement with recent reports showing the association of NMDA stimulation in formalin-induced pain [60, 61]. Treatment with the NMDA receptor-antagonist AP5 produced analgesia in a formalin pain test [62]. Although our experimental design called for a different time course and route of stimulation than the formalin pain test, the phenomenon of NMDA receptor activation is a common response to FA.

It is suggested that the interaction between the neural and immune systems is an underlying cause of MCS. We conducted an experiment to examine the effects of FA exposure in OVA-immunized mice [63]. In that study, the mice (C3H females) were injected intraperitoneally with 10 *μ*g OVA and 2 mg alum before their exposure to FA (0 or 400 ppb). On days 21, 42, 63, and 77 of the exposure period, each mouse was booster challenged with OVA, and the brains were sampled one day following the final FA inhalation. To determine the effects of low-level FA exposure on the expression of neuronal synaptic plasticity related genes in the hippocampus, we examined the mRNA expression of the NMDA receptor-subtypes NR2A and NR2B, dopamine D1 and D2 receptors, cAMP response element-binding (CREB)-1 and -2, and FosB/ΔFosB. The mRNA levels of NR2A, dopamine D1 and D2 receptors, and CREB-1 significantly increased in 400 ppb FA-exposed mice compared to the control mice, but the levels of NR2B, CREB-2, and FosB/ΔFosB were not changed [63]. We also found that treatment with MK-801, a noncompetitive NMDA receptor antagonist, normalized the NR2A, CREB-1, and dopamine D1 and D2 receptor mRNA levels that were induced by FA exposure [63]. These results suggest that stimulation by low-level FA exposure and OVA immunization selectively affects synaptic plasticity-related genes in the hippocampus and that the effects are mediated by glutamatergic neurotransmission through NMDA receptors. 

To the best of our knowledge, our serial reports showing that exposure to low levels of FA alone can alter the molecular basis of brain neural transmission and that a combination of OVA immunization and FA exposure induces a significant increase in hippocampal NMDA receptor mRNA levels. It is well known that NMDA receptors are a key molecule for the formation of neuronal memory. Because neuronal memory formation is a major phenomenon in neural sensitization, and neuroimmune interaction is thought to participate in the development of MCS, it follows that the increase in hippocampal NMDA after OVA immunization and FA exposure reflects, at least in part, the hypersensitivity underlying MCS.

We previously described the effects of FA on the expression of the Bcl-2 family, which regulate survival and death of cells, and the NMDA receptors, which are associated with hippocampal function, in mice [64]. In that study, Western blot analyses were performed for Bcl-2, Bax, NMDA receptors types 2A and 2B (NR2A and NR2B) of the hippocampus taken from C3H mice exposed to 0 or 400 ppb of FA with or without OVA immunization [64]. We found that the ratio of Bcl-2/Bax expression in OVA-immunized mice significantly increased with 400-ppb FA exposure, although the differences in the expression level of each protein were not significant between the control and FA-exposed groups. The NR2A and NR2B expression of FA-exposed OVA immunized mice were sustained at levels comparable to the control; however, there was no difference in NR2A, NR2B expression, or Bcl-2/Bax expression ratio in mice that were not OVA immunized and exposed to 400 ppb FA [64]. These results suggest that the changes in the Bcl-2/Bax expression ratio that occur with low-level FA exposure and OVA immunization may follow enhanced NGF production and exert a protective effect against apoptosis. We speculate that our putative defense mechanisms are initially exerted in the hippocampus in response to FA exposure and that adverse FA effects successively arise when the FA exposure reaches an intolerable level. This mechanism does not account for the transition of the influence of FA from “protective” to “adverse”; however, it may be a key for understanding FA sensitivity in individuals that complain of unexplained symptoms following extremely low-level FA inhalation.

## 7. Conclusion

**Figure 1:**
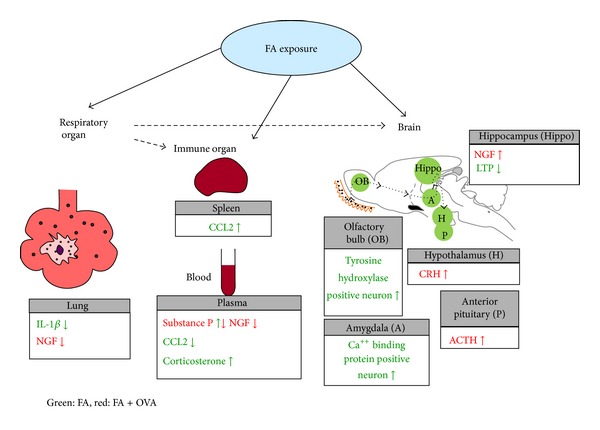
Possible target organs and biomarkers affected by formaldehyde exposure. FA exposure with or without OVA affects respiratory, immune, and central nervous systems by modulating the cytokines (IL-1*β*, CCL2), neuropeptides (NGF, substance P), hormones (CRH, ACTH, and corticosterone), and enzymes (TH) and intracellular calcium-binding protein in the mouse model (green: FA, red: FA + OVA).

Long-term exposure to low levels of FA can disturb normal homeostatic responses by enhancing a neural network that is abnormally activated by coexposure to immunological stimuli and results in the induction of neurogenic and immunogenic inflammation in the brain. These responses may be related to the triggering of MCS. Possible target organs and biomarkers affected by formaldehyde exposure with or without OVA were shown in Figure 1.
